# Implementing national COVID-19 vaccination programmes in sub-Saharan Africa- early lessons from Zimbabwe: a descriptive cross-sectional study

**DOI:** 10.11604/pamj.2021.40.180.30824

**Published:** 2021-11-24

**Authors:** Grant Murewanhema, Trouble Victor Burukai, Brighton Chireka, Edward Kunonga

**Affiliations:** 1 Unit of Obstetrics and Gynaecology, Faculty of Medicine and Health Sciences, University of Zimbabwe, Harare, Zimbabwe,; 2 Zimbabwe College of Public Health Physicians, Harare, Zimbabwe,; 3 Department of Anatomy, Faculty of Medicine and Health Sciences, Midlands State University Harare, Harare, Zimbabwe,; 4 School of Health and Life Sciences, Teesside University, Middlesbrough, United Kingdom

**Keywords:** COVID-19, pandemic, vaccination, vaccines, hesitancy, Zimbabwe, sub-Saharan Africa

## Abstract

**Introduction:**

Zimbabwe was one of the first countries to run a national COVID-19 vaccination programme in Africa. Lessons learnt could inform the roll-out of similar programmes in sub-Saharan Africa. To describe the trends of uptake of the COVID-19 vaccines in the first three months (February - May 2021) of the Zimbabwe vaccination programme and the lessons learnt.

**Methods:**

a secondary descriptive analysis of routinely available COVID-19 vaccination data extracted from the daily situation reports published by the Ministry of Health and Child Care.

**Results:**

in the first three months of the programme, 1 020 078 doses were administered, with 675 678 being first doses and 344 400 were second doses. Using population estimates, at three months, 5.2% of the population had received at least one dose and 2.6% had received the full two doses. Uptake was initially slow, followed by a gradual, and subsequently an exponential increase.

**Conclusion:**

by the end of May 2021, Zimbabwe had rolled out one of the largest COVID-19 vaccination programme in sub-Saharan Africa. The uptake followed a pattern and trend that is consistent with vaccine hesitancy reported in the literature, driven by a combination of confidence, complacency and convenience factors. The gradual increase in uptake followed a series of national and local community engagement programmes. The roll-out of similar programmes must recognise likely patterns of uptake across the population and ensure plans are in place to address vaccine hesitancy. The available data did not allow granular analysis to understand the demographics of people who participated in the programme, which is important for surveillance, targeted action, preventing inequalities and ensuring adequate and proportionate protection of residents prioritising the most vulnerable. Further analysis of the process, outcomes and impact of the programme will be helpful in informing the roll-out of similar programmes across Africa.

## Introduction

An unusual cluster of pneumonia was detected in Wuhan in the Chinese Province of Hubei around December 2019 [1]. The outbreak rapidly evolved into an international public health emergency, [2] and declared a pandemic by the World Health Organization (WHO) in March 2020 [3]. Global health efforts to contain the pandemic included travel restrictions and national lockdowns. Despite the control efforts, the spread of the severe acute respiratory syndrome Coronavirus 2 (SARS-CoV-2), the causal agent of the disease now known as Coronavirus Disease 2019 (COVID-19) continued inexorably. Some countries are now in a third wave, and the majority of countries have experienced at least a second wave. The latest WHO situation reports indicate that as of 29^th^ June 2021, an estimated cumulative 180 492 131 cases of COVID-19 had been confirmed globally, with 3 916 771 fatalities, and furthermore, an estimated 2 600 313 cases had been reported in the preceding seven days [4]. Therefore, significant numbers of incident COVID-19 cases continue to be reported. The Americas, Europe and East Asia continue to report the highest numbers of cases and fatalities from COVID-19 [4]. However, Africa, which was initially projected to be decimated by the pandemic [5] has continued to report fewer cases and fatalities, with a cumulative 3,968 421 cases, 94 323 deaths and 177 367 incident cases in the seven days prior to 29^th^ June 2021 [4]. The trends of the epidemic in Zimbabwe largely mirror the rest of the African continent, with less than 60,000 cumulative cases and less than 2000 fatalities to date [6]. The current epidemic curve reveals a third wave from the beginning of June 2021 after the harsh second wave experienced between December 2020 and February 2021 [6]. The crux of COVID-19 prevention since the start of the pandemic was premised on human-dependent infection prevention and control measures, including physical distancing, wearing face masks, washing hands frequently, and lockdowns imposed by governments.

However, these were not enough, as evidenced by the continued spread of the virus and loss of lives. The emergency use authorization (EUA) of vaccines by the WHO [7] brought hope of another layer of protection to potentially limit the further spread of SARS-CoV-2 and limit the morbidity and mortality associated with a rapidly spreading infectious disease. Vaccination is one of public health´s greatest success stories, and has drastically reduced the morbidity and mortality associated with some infectious diseases [8]. However, population level benefits of vaccination will be realized after fully vaccinating a proportion significant enough for herd immunity to be attained, estimated to be about 67% for COVID-19 [9,10]. Several SARS-CoV-2 vaccines have been formulated, including mRNA-based, viral vector based and inactivated viral vaccines [11] however, not all of them have obtained WHO EUA. The interval from pandemic onset to successful roll-out of these vaccines has been dramatic, premised on experiences from years of vaccinology and immunology research. The global vaccination program has faced challenges since its inception. Vaccine hesitancy is a big threat to success [12] compounded by various conspiracy theories regarding the origins of COVID-19, nationalization of COVID-19 vaccines, inequitable distribution, and global supply chain challenges [10,11]. The low and middle income countries may be the worst affected by these factors [11]. The WHO defines vaccine hesitancy as refusal to be vaccinated or delays in being vaccinated despite the availability of vaccines [13]. Several factors contribute to vaccine hesitancy, and have been broadly categorized into complacency, confidence and convenience factors [13]. Adequate vaccination uptake in any population involves adequately dealing with promoters of vaccine hesitancy, and taking advantage of the factors that promote vaccine confidence and uptake [10].

The other challenge in the global COVID-19 vaccination efforts may be the supply of vaccines in an equitable way. Richer, more powerful countries/economies, may secure larger shares of vaccines as they prioritize vaccinating their citizens, disadvantaging resource-challenged settings [11]. Adequate safeguards are required to ensure equitable distribution of vaccines, and leave no country, region or continent with a huge burden of an unvaccinated population [7]. Zimbabwe was not a part of the COVAX arrangement, and had no access to vaccines that were included in the arrangement thereof. The government coordinated the country´s access to COVID-19 vaccines mainly from China, India and lately Russia. The first batch of vaccines consisted of a donation of 200 000 Sinopharm vaccines from China on 15^th^ February 2021 [14]. This led to the official launch of the Zimbabwe national COVID-19 vaccination program on 18^th^ February 2021 [15] and for it to follow the staged approach that prioritized frontline workers and people vulnerable due to old age and/or chronic conditions (Table 1) [16]. Zimbabwe was one of the countries that made significant strides in initiating the vaccination program despite several challenges, and successfully started the program ahead of its many other counterparts. Therefore, substantial lessons can be derived from the Zimbabwean program, which may help other countries in the region and beyond to shape and refine their vaccination roll-out. Ongoing surveillance of the program to take stock of uptake must go on a continual basis. The objective of this study was to examine the trends of vaccination in Zimbabwe since the beginning of the program, based on publicly available data from daily situational reports and discuss barriers and promoters of uptake in order to adequately inform public health policy and strategy.

**Table 1:** COVID-19 roll-out phases planned by the Ministry of Health and Child Care of Zimbabwe

Phase	Targeted vaccine recipients
**1**	Stage1: targeted frontline workers including healthcare workers, ports of entry personnel, immigration, customs and security.
	Stage 2: targeted people with chronic illnesses, the elderly (60 years and above), prison inmates and others in confined settlements.
**2**	Lecturers, schoolteachers and other staff at medium risk depending on the epidemiological picture of the disease.
**3**	Population at low risk.

## Methods

**Study design:** this was a retrospective cross-sectional analysis of vaccination data that was made publicly available by the Ministry of Health and Child Care of Zimbabwe (MOHCC) through its daily situational reports.

**Study setting, participants and data sources:** data were extracted from the daily situation reports for a period from 18^th^ February 2021 to May 2021. The MOHCC of Zimbabwe releases daily reports containing national COVID-19 statistics since the onset of the pandemic, including incident, cumulative, recovered and active COVID-19 cases, fatalities as well as COVID-19 vaccination data. This was an analysis of aggregated data; therefore, no individual participants were included.

**Variables:** the only variables included in the study were the number of people who had received the first dose of any COVID-19 vaccine being administered in Zimbabwe, (namely Sinopharm, Sinovac and Covaxin vaccines), those who had received the second dose, and those who had received both doses, considered as fully vaccinated.

**Bias:** potential sources of bias for this study include missing data, and incomplete reporting by the MoHCC. However, no significant impact was expected from both, and because this was just a descriptive analysis of routinely available data, there were no adjustments and no inferential statistics. The inherent weaknesses of such data are noted; however, it is anticipated that significant lessons can still be obtained from the observed vaccination trends.

**Study size:** all the available COVID-19 vaccination data available from the daily situation reports were extracted and included in the analysis. No sample size calculations were required.

**Statistical methods:** no advanced inferential statistics or models were done for this set of vaccination data in this descriptive cross-sectional study. Appropriate graphs were generated in Stata Version 16.0.

**Ethical considerations:** this was a secondary analysis of publicly available data, and no ethical approvals were necessary. Since no individual participant data were collected, no individual informed consents were sought.

## Results

**Figure 1:**
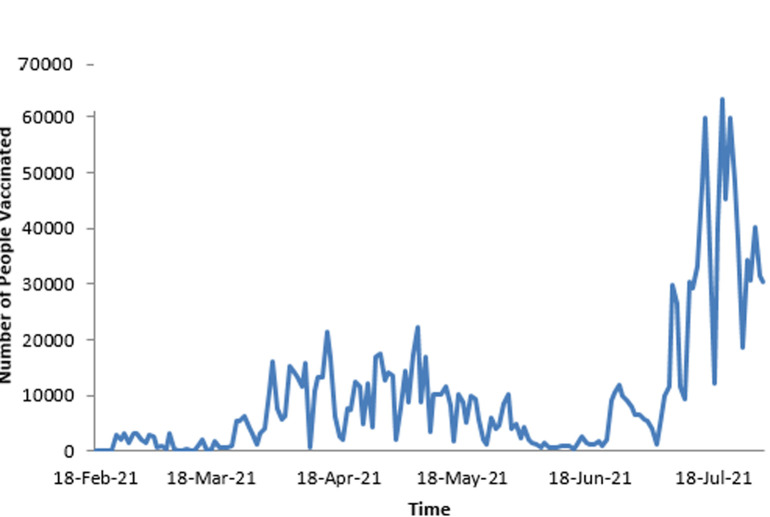
the daily uptake of the first dose of COVID-19 vaccines during the first three months of roll-out in Zimbabwe

**Figure 2:**
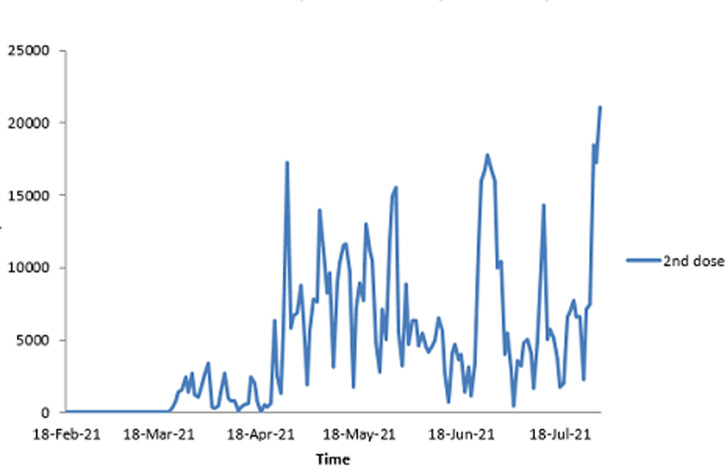
the daily uptake of the second dose of COVID-19 vaccines during the first three months of uptake

**Figure 3:**
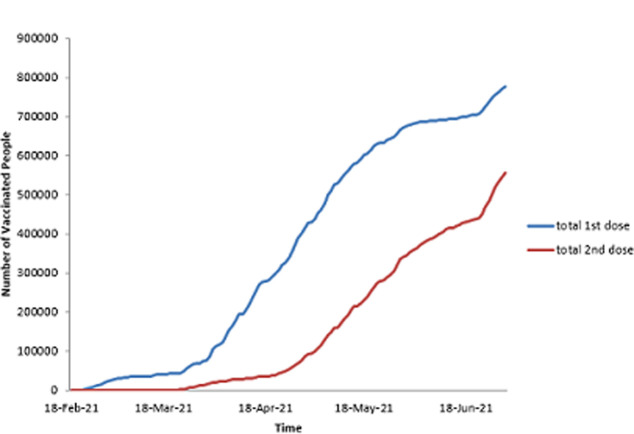
the cumulative uptakes of the first and second doses of COVID-19 vaccines in Zimbabwe during the first three months of roll-out

As stated previously, the first dose of SARS-CoV-2 vaccine was administered on 18^th^ February 2021. As shown in Table 2, by the end of the first month (March 2021), 76 995 people had received the first dose and 14 885 received the second dose. The figures increased by the end of third month to 675 678 for the first dose and 344 400 for the second dose, representing 5.2% and 2.6% of the population respectively. Figure 1 shows the daily uptake of the first dose for the first three months. It was low at the beginning with less than 5000 doses administered daily, almost came to a halt around 12 March 2021, and then started rising again, reaching a peak by mid-April 2021 with a highest of over 20 000 doses in one day. However, there are significant, notable gaps throughout the whole period when very low numbers of doses were administered. The two doses were spaced a month apart. Figure 2 shows the uptake of the second dose since the beginning of the program, and shows a pattern that largely resembles that of the first dose. Figure 3 illustrates the cumulative uptake of the vaccines and shows the initial slow trajectory for most of the first month, followed by an exponential increase in uptake in the second and third months of roll-out.

**Table 2:** COVID-19 vaccination statistics during the first three months of roll-out

Period	People who received the first dose (%Population)	People who received the second dose (%Population)
1st month - end of March 2021	76 995 (0.589%)	14 885 (0.114%)
2nd month - end of April 2021	414 735 (3.175%)	85 607 (0.655%)
3rd month - end of May 2021	675 678 (5.173%)	344 400 (2.637%)

## Discussion

The roll-out of the COVID-19 vaccines has been highlighted as a significant development in the war against the pandemic globally [7]. There have been significant concerns raised with the roll-out of the vaccination programmes regarding vaccine nationalism with inequitable access to the vaccines globally [7,11]. By the end of May, almost 1 billion doses of the vaccine had been administered globally, but less than one percent of these were in developing countries [17]. The majority of low and medium income countries were very slow to have access to vaccines to administer to their local populations [11]. Zimbabwe was one of the first countries to announce a very ambitious programme. The government pledged to acquire a million doses of vaccines monthly to vaccinate the population in phases, with prioritisation of frontline healthcare workers and other people in high-risk jobs [16]. The country received its first batch of 200 000 Sinopharm vaccines on 15^th^ February 2021 [14], and subsequently received further doses of Sinopharm, Sinovac and Covaxin vaccines. The Medicines control a of Zimbabwe (MCAZ) announced its approval of the Sinopharm vaccine for emergency use in the country in February 2021 and subsequently authorised the use of Sinovac, Covaxin and Sputnik vaccines [18]. At this time, these vaccines had not obtained EUA by the WHO. This played a role in the vaccine concerns and hesitancy in the population. The authors experienced this in the community awareness sessions where concerns were raised regarding participating in a vaccination programme that was not endorsed and did not have effectiveness and safety data publicly available as well as a wider mistrust of Chinese products based on experience with other products and commodities. Sinopharm received WHO approval on the 7^th^ of May 2021 joining Pfizer/BioNTech, Astrazeneca-SK Bio, Serum Institute of India, Janssen and Moderna vaccines that had received earlier approval for emergency use [19]. The data on vaccine uptake obtained from the MOHCC´s daily situation reports demonstrated a slow start in uptake of both the first and second doses in the first month. This is in line with the authors´ personal experiences of the levels of concerns people had regarding the safety and effectiveness of the vaccines.

Trends show a very slow initial uptake of both the first and second doses. Unlike the pattern of uptake in western countries, the slow uptake is in line with the concerns that have been reported in a number of studies on vaccine concerns and vaccine hesitancy in African countries [20,21]. Perhaps not surprising this pattern is similar to that observed in Africans living in the United Kingdom (UK). Studies have shown poor uptake of COVID-19 vaccines amongst Black Asian and minority ethnic (BAME) groups in the UK with confidence in the safety and effectiveness of the vaccines being some of the most significant issues [22,23]. A combination of misinformation, mal-information and disinformation has played a significant role in the poor initial uptake of the vaccine. It has been reported that a number of conspiracy theories have circulated in these communities mainly on social media and have not been consistently matched with genuine and accurate information on the vaccine [24]. If countries are to have a successful roll out of national COVID-19 vaccination programmes work is required to engage communities and raise awareness as well as address the concerns that people might have on the vaccine safety and effectiveness [10]. The Zimbabwe COVID-19 vaccination programme reported a sharp rise in the number of doses administered in the second and third months. There are a number of possible explanations for this rapid rise in uptake. Zimbabwe has a strong expanded programme of immunisation (ZEPI) that has been successful over the years and provided important infrastructure to rapidly mobilise the roll-out of the national COVID-19 vaccination programme [25]. There was a wide range of awareness raising campaigns delivered by a wide range of stakeholders including the government, private sector, non-governmental organisations and civil society. This combined with the gradual increase in uneventful vaccinations generated sufficient numbers for people to grow in confidence and interest in the vaccine and the vaccination programme. Vaccine complacency has been widely acknowledged and the importance of seeing leaders, health and care professionals, peers, family and friends being vaccinated on confidence and acceptance [10,26].The concept of using peer educators and peer support arrangements is a well-established phenomenon in the success of multi-component public health programmes and may have played an important role in increasing uptake in Zimbabwe. There was remarkable press and social media coverage of high profile figures such as politicians [27], celebrities and health professionals [28] being seen publicly receiving the vaccinations. Health and care workers have an important role in increasing vaccine uptake for COVID-19 and any other vaccinations. This was reported widely in the UK where health and care workers from BAME communities were involved in promoting the vaccine and running a number of community sessions [23,29].

In Zimbabwe, however, there were initially high levels of vaccine hesitancy amongst health and care workers [30]. This led to the general population having concerns regarding the vaccines. With the need to have sufficient numbers to increase confidence, health and care workers were given a deadline beyond which the vaccination programme was opened up to the general population. It is not clear from the publicly available data, the uptake of the vaccines amongst health and care workers. The decision to open up the vaccines to the rest of the population might have helped to increase the number of people who received the vaccines in Zimbabwe during the first three months of the national programme. Whilst this may have helped in increasing confidence in the vaccine, it is not possible to assess whether this increase was from the priority and target groups. The publicly available information did not present uptake by priority group or vulnerability to align with the staged approach to the roll-out of the vaccination programme. As part of surveillance, and to enable targeted action and protecting the most vulnerable people first, it will be very useful for the vaccine uptake to be reported by these subgroups as the characteristics of those who participated in the programme. This is important to afford the vaccination programme widening inequalities by geography (urban vs rural), ethnicity, social status, occupation, age and other key considerations that are related to access as well as vaccine hesitancy. Data regarding distribution of the vaccines throughout the country is not available; however, reports indicate that initial vaccination was concentrated in the big cities, starting with Harare. It is possible that people elsewhere may have been willing to be vaccinated, but the vaccines were not accessible to them. Uptake of public health programmes depends on accessibility, with a population that is socioeconomically deprived unlikely to travel long distances to access services. COVID-19 control travel restrictions were still also in place when the vaccination programme commenced. Equitable distribution of vaccines is necessary, especially as the most vulnerable groups in the population may have no easy access to the vaccines, requiring effective strategies to ensure reach. There was variation in performance of the vaccination programme with days when little or no vaccines were administered. This could have been due to weekend variation in operating times, supply chain disruptions and logistical challenges in getting the vaccines to points of administration, but also possibly days of heightened hesitancy by the population. There were some media scares on the impact on vaccinations with one ending up in the mainstream media of someone who had died following vaccination [31]. The government issued an immediate press release to address this issue; however, social media misinformation was high around that time. Local drivers of vaccine hesitancy have not been adequately explored and addressed in full although there are emerging research papers and policy positions on this issue in Zimbabwe [30]. Vaccine hesitancy has been described as 'pervasive, misinformed, contagious, and is not limited to COVID-19 vaccination´ [32]. Addressing vaccine hesitancy requires an understanding of its complexity, the key concerns people have and the context in which people make decisions about their health, healthcare and well-being

The drivers of COVID-19 vaccine hesitancy have been described as being based on myths, misconceptions and conspiracy theories that have largely been circulated in the social media, with a wide population reach [31]. Influential and popular religious leaders and antivaxxers have largely peddled messages concerning the vaccines as the mark of the beast, the antichrist, a way to exterminate the population, and metamorphosis of the vaccinated into aliens [29]. The power of such should never be underestimated. Other circulating misconceptions include the alteration of human DNA by the vaccines and the injection of live COVID-19 virions [33]. Of concern in the reproductive age groups has been the rumours that the vaccines cause infertility, and safety concerns regarding pregnancy and breastfeeding, possibly propagating hesitancy among women of reproductive age [34]. Zimbabweans, like other Africans, may have also been suffering from a preventive misconception from some forces [35], promoting vaccine hesitancy. Scholarly projections by a team from the London School of Hygiene and Tropical Medicine had earlier on projected that Africa would suffer the greatest from the direct and indirect effects of the COVID-19 pandemic [5]. However, several months later, the African continent is still among the least hit in terms of absolute cumulative numbers and case fatalities, and recovery statistics have been very impressive [4]. Both the first and second waves of the pandemic did not have the projected devastating effects on the continent [36], and Africans have believed they are somehow immune to COVID-19, with several hypotheses having been put forward but never verified. Mistrust around the expedited vaccine development has emerged in a continent where the HIV epidemic has been going on for four decades without safe and effective vaccines. Building trust, maximising convenience and accessibility and ensuring uninterrupted supply chains are essential components for the success of the vaccination programme. As the country plunges into a third wave, the government is faced with a mammoth task of ensuring that we have vaccinated the population to levels sufficient to attain herd immunity. With less than 5% of the population fully vaccinated, this is an uphill task, and therefore urgent public health interventions to optimise vaccine uptake whilst we practise the WHO prevention strategies are warranted.

**Limitations:** this was a descriptive analysis of publicly available vaccination data, subject to missing data and incomplete recording. The data did not allow for granular analysis of vaccination trends by demographic characteristics such as age, gender, province of origin, religion, socioeconomic status, level of education and racial profiles. Such an analysis would have allowed for targeted interventions to optimise vaccination uptake among different populations. **Generalisability:** important lessons have been described as above; however, these may not be generalised to other contexts, as different factors might come into play to influence vaccine uptake, such as religious beliefs, socioeconomic status and level of education, which were not available for this analysis.

## Conclusion

Zimbabwe administered almost 1million doses of COVID-19 vaccines to its population in the first three months of the national vaccination programme. This was made possible by rapid access and approval for the use of Sinopharm, Sinovac and Covaxin vaccines. The roll out of the vaccines was characterised by three phases: slow start, rapid growth and sustained high levels in the first three months respectively. These stages seem to be driven by the levels of vaccine confidence and acceptance as well as trust in the vaccine. The routinely available data on uptake of the vaccination programme in Zimbabwe does not allow further analysis of the differential in uptake that might exist by vulnerability and other demographic and socioeconomic factors. This is very important to provide assurance of equitable and proportionate roll out of the programme that should prioritise the protection of the most vulnerable first and fast. The roll out of the vaccination programmes in sub-Saharan Africa will benefit from the lessons learnt from Zimbabwe and plan for the pattern of uptake in their countries. As the time moves on and a lot becomes known about the COVID-19 disease, the virus and vaccines, it is possible that other countries will not follow the same pattern in the uptake of vaccines. Further studies are required to further understand the roll-out of the Zimbabwe COVID-19 vaccination programme as it continues, as well as other countries whose programmes are in the initial phases.

### What is known about this topic

- Vaccination is the most effective public health intervention to limit the spread, morbidity and mortality associated with rapidly spreading infectious diseases;
- The uptake of vaccines can be influenced by convenience, complacency and confidence factors;
- Vaccine hesitancy can be a serious threat to the uptake of COVID-19 vaccines in any population.

### What this study adds

- Zimbabwe was one of the first countries to run a national COVID-19 vaccination programme in Africa, delivering over one million doses to its population in the first three months;
- The uptake of the vaccines was initially slow, followed by a gradual, then an exponential increase in uptake;
- The uptake followed a trend of vaccine hesitancy that is similar to what has been described in the literature.
